# *B. Abortus* Modulates Osteoblast Function Through the Induction of Autophagy

**DOI:** 10.3389/fcimb.2018.00425

**Published:** 2018-12-04

**Authors:** Ayelén Ivana Pesce Viglietti, Maria Virginia Gentilini, Paula Constanza Arriola Benitez, Guillermo Hernán Giambartolomei, María Victoria Delpino

**Affiliations:** Instituto de Inmunología, Genética y Metabolismo (INIGEM), Universidad de Buenos Aires, Consejo Nacional de Investigaciones Científicas y Técnicas, Buenos Aires, Argentina

**Keywords:** *Brucella*, bone, osteoblast, autophagy, osteoblast activity

## Abstract

Osteoarticular brucellosis is the most common localization of human active disease. Osteoblasts are specialized mesenchymal-derived cells involved in bone formation and are considered as professional mineralizing cells. Autophagy has been involved in osteoblast metabolism. The present study demonstrates that *Brucella abortus* infection induces the activation of the autophagic pathway in osteoblast cells. Autophagy was revealed by upregulation of LC3II/LC3I ratio and Beclin-1 expression as well as inhibition of p62 expression in infected cells. Induction of autophagy was also corroborated by using the pharmacological inhibitors wortmannin, a PI 3-kinase inhibitor, and leupeptin plus E64 (inhibitors of lysosomal proteases). Autophagy induction create a microenvironment that modifies osteoblast metabolism by the inhibition of the deposition of organic and mineral matrix, the induction of matrix metalloproteinase (MMP)-2, osteopontin, and RANKL secretion leading to bone loss. Accordingly, autophagy is also involved in the down-modulation of the master transcription factor in bone formation osterix during *B. abortus* infection. Taking together our results indicate that *B. abortus* induces the activation of autophagy pathway in osteoblast cells and this activation is involved in the modulation of osteoblast function and bone formation.

## Introduction

Bacteria of the genus *Brucella* are Gram-negative microorganisms that infect domestic and wild animals and produce a weakening chronic disease with participation of multiple organs in human patient (Pappas et al., 2005). Brucellosis commonly results in persistent, chronic involvement of osteoarticular system which usually leads to bone damage (Young, 1989; Madkour, 2001; Aydin et al., 2005; Colmenero et al., 2008).

The synchronized action of osteoclasst, osteoblasts, and osteocytes are necessary for the maintenance of skeletal homeostasis. Autophagy pathway has been recently implicated in bone homeostasis as was revealed by the presence of proteins involved in autophagic protein degradation as mediators of osteoclast, osteoblast, and osteocyte function, suggesting new pathogenic mechanisms in bone disease (Nollet et al., 2014).

Autophagy is a lysosome-based recycling pathway that disassembles unnecessary or dysfunctional components in order to promote cell survival and function, especially under stressful environment (Nollet et al., 2014). During this well-established mechanism protein aggregates or old organelles became engulfed by a double membrane vesicle called autophagosome that fuses with lysosomes with concomitant degradation of its contents (Liu et al., 2013). Thereby, autophagy provides further sources of energy and helps cells to remove injured organelles such as mitochondria, both of which support the survival and function of cell. Autophagy is significant in long-lived cells and a decline in autophagy has been proposed as a justification for the changes that take place in degenerative diseases (He et al., 2013). Significantly, It has been demonstrated that deletion of genes related with autophagy such as Atg7 completely suppresses the course of autophagy, allowing researchers to examine the significance of this pathway in various cell types (Mizushima et al., 2010).

Osteoblasts are mesenchymal-derived cells that are responsible for bone formation through the synthesis and secretion of bone matrix, and its subsequent mineralization. These large quantities of bone matrix are under strict control to support rapid protein synthesis and avoid secretion of misfolded matrix proteins. Autophagy is involved in osteoblast activity and differentiation (Nollet et al., 2014). Studies using chloroquine and 3-methyladenine revealed that autophagy is required for osteoblast terminal differentiation, but not in initial stages of differentiation (Liu et al., 2013). Disruption of autophagy could result in loss of bone mineral density and bone mass because of decreased osteoblast differentiation and activity. But, the importance of autophagic flux for bone matrix secretion and the role of autophagy in osteoblast function during bacterial infection remain unknown.

We have previously demonstrated that *B. abortus* can infect and survive within human osteoblasts and that this infection induces the expression of chemokines, proinflammatory cytokines, and matrix metalloproteases (MMPs), that could be implicated in the osteoarticular manifestations of brucellosis. Also *Brucella* infection inhibits osteoblast differentiation and function (Scian et al., 2012). Thus, we hypothesized that *Brucella* infection would generate a microenvironment that promotes the modulation of osteoblast function and induces activation of autophagic pathway, which might have an important contribution in bone damage during osteoarticular brucellosis.

## Materials and Methods

### Bacterial Culture

*Brucella abortus* S2308 and *Brucella abortus* RB51, a stable rough mutant, were grown overnight in 10 ml of tryptic soy broth (Merck, Buenos Aires, Argentina) with constant agitation at . Bacteria were harvested and the inocula were prepared as described previously (Scian et al., 2011). All live *Brucella* manipulations were performed in biosafety level 3 facilities located at the Instituto de Investigaciones Biomédicas en Retrovirus y SIDA (INBIRS).

### Cellular Infection

Osteoblast cell line MC3T3-E1 was infected with *B. abortus* at different multiplicities of infection (MOI). After the bacterial suspension was dispensed, the plates were centrifuged for 10 min at 2,000 rpm and then incubated for 2 h at under a 5% CO_2_ atmosphere. Cells were extensively washed with DMEM to remove extracellular bacteria and incubated in medium supplemented with 100 μg/ml of gentamicin and 50 μg/ml of streptomycin to kill extracellular bacteria.

MC3T3-E1 cells were harvested at different times (see below), to determine the induction of molecules involved in autophagy and how this pathway affect cellular differentiation and function.

### Western Blotting

Infected MC3T3-E1 cells were lysed in ice-cold lysis buffer consisting of 20 mM HEPES (pH 8), 5 mM EDTA, 10 mM EGTA, 5 Mm NaF, 10% glycerol, 1 mM dithiothreitol, 400 mMKCl, 0.4% Triton X-100, 20 mM sodium glycerophosphate, and a protease inhibitor cocktail (Sigma-Aldrich). Lysates were incubated on ice for 10 min and cleared by centrifugation at 13,000 g for 10 min. Protein concentration was determined by the bicinchoninic acid method (Pierce, Rockford, IL, USA) using bovine serum albumin as standard, and equal amounts of proteins were loaded onto electrophoresis gels. After separation, proteins were transferred to a nitrocellulose membrane (GE Healthcare, Little Chalfont, UK) and blocked for 1 h with 5% milk protein-0.05% Tween 20. Then, membranes were incubated with rabbit anti-MAP LC3B (Cell signaling), goat anti-BECN-1 or anti-p62 (Santa Cruz Biotechnology, Santa Cruz, CA) overnight at 4°C. After washing, the membrane was incubated with peroxidase-conjugated secondary antibody (Santa Cruz Biotechnology, Santa Cruz, CA) (1:1,000 dilution), for 1 h. Protein bands were visualized on Hyperfilm ECL (GE Healthcare) by chemiluminescence. Equal loading was checked by Ponceau staining and by incubation of the blots with an anti-actin (clone C4; Santa Cruz Biotechnology).

### Measurement of Cytokine Concentrations

Secretion of Receptor activator of nuclear factor-κB ligand (RANKL) (R&D systems) was quantified by ELISA from in culture supernatants.

### Inhibition of Autophagy

To study the potential involvement of autophagy pathways in calcium deposition, collagen deposition, MMP-2, RANKL secretion by *B. abortus* infection in MC3T3-E1 cells, wortmannin or E64 plus leupeptin, bafilomycin A1, or chloroquine (Sigma-Aldrich de Argentina S.A.) were added 2 h before the beginning of infection. Wortmannin was used at a concentration of 10 μM, E64 plus leupeptin were used at a concentration of 20 μM, Bafilomycin A1 was used at a concentration of 200 nM and Chloroquine (Sigma-Aldrich de Argentina S.A.) was used at a concentration of 50 μM (Mizushima et al., 2010).

### Zymography

Gelatinase activity was assayed by the method of Hibbs et al. with modifications, as described (Hibbs et al., 1985; Scian et al., 2011).

### Osteoblast Differentiation

Osteoblast differentiation involves the expression of genes implicated in the specific function of osteoblasts to secrete mineral and organic matrix. To elucidate this, we studied calcium and collagen, deposition. To this end, cultures of osteoblast cells were seeded onto glass coverslips and were infected with *B. abortus* as described above.

Experiments with recombinant human bone morphogenic protein (rBMP)-2 (Sigma-Aldrich de Argentina S.A) were performed by adding 10 ng/ml of rBMP-2 during osteoblast differentiation.

### Assessment of Collagen Deposition by Sirius Red Staining

Collagen deposition was quantified by using Sirius red (Sigma-Aldrich de Argentina S.A.), a strong anionic dye that binds strongly to collagen molecules (Tullberg-Reinert and Jundt, 1999). Sirius red was dissolved in saturated aqueous picric acid at a concentration of 0.1%. Bouin's fluid (for cell fixation) was prepared by mixing 15 ml saturated aqueous picric acid with 5 ml 35% formaldehyde and 1 ml glacial acetic acid. Cell layers were extensively washed with PBS before they were fixed with 1 ml Bouin's fluid for 1 h. The fixation fluid was removed and the culture plates were washed 3 times with deionized water. The culture dishes were air dried before adding 1 ml Sirius red dye reagent. The cells were stained for 18 h with mild shaking. The stained cell layers were extensively washed with 0.01 N hydrochloric acid to remove all unbound dye. After rinsing, coverslips were mounted in PBS-glycerine (9:1 [vol/ vol]) and were analyzed by light microscopy. For quantitative analysis, the stained material was dissolved in 0.2 ml 0.1 N sodium hydroxide by shaking for 30 min. The dye solution was transferred to microtiter plates, and the optical density (OD) measured with a microplate reader (Metertech, Inc., Taiwan) at 550 nm against 0.1 N sodium hydroxide as a blank.

### Assessment of Calcium Deposition Alizarin Red S Staining

To determine calcium deposition, we used alizarin red staining. Osteoblast cells seeded onto glass coverslips were infected with *B. abortus* as described above. On day 7 of the culture's differentiation, osteoblasts were fixed in 4% PFA for 10 min at room temperature. The cells were washed with deionized water and stained with 2% (wt/vol) alizarin red S and were visualized by light microscopy or extracted to perform quantitative analysis.

### mRNA Preparation and qPCR

RNA was extracted using the Quick-RNA MiniPrepKit (Zymo Research), and 1 μg of RNA was subjected to reverse transcription using Improm-II Reverse Transcriptase (Promega). PCR analysis was performed with StepOne™ Real-Time PCR System (Applied Biosystems) using SYBR Green as fluorescent DNA binding dye. The primer sets used for amplification were as follows: β-actin sense: 5′-AACAGTCCGCCTAGAAGCAC-3′, antisense: 5′-CGTTGACATCCGTAAAGACC-3′; Runt-related transcription factor 2 (Runx2) sense: 5'-TGCACCTACCAGCCTCACCATAC-3', antisense 5'- GACAGCGACTTCATTCGACTTCC-3'; osteopontin (OPN) sense 5'- TTCACTCCAATCGTCCCTAC-3', antisense: 5'- TGCCCTTTCCGTTGTTGTC-3'; Osterix (Osx) sense 5'- AGCGACCACTTGAGCAAACAT-3', antisense: 5'- GCGGCTGATTGGCTTCTTCT-3'

The amplification cycles for Runx2 and OPN were 95°C for 15 s, 56°C for 30 s and 72°C for 60 s; for Osx and β-actin were 95°C for 15 s, 60°C for 30 s and 72°C for 60 s. All primer sets yielded a single product of the correct size. Relative expression levels were normalized against β-actin.

### Statistical Analysis

Statistical analysis was performed with one-way ANOVA, followed by *Post Hoc* Tukey Test using GraphPad Prism 5.0 software. Data were represented as mean ± SD.

## Results

### *B. abortus* Infection Induces LC3-II and Beclin-1 Expression in MC3T3-E1 Cells

**Figure 1:**
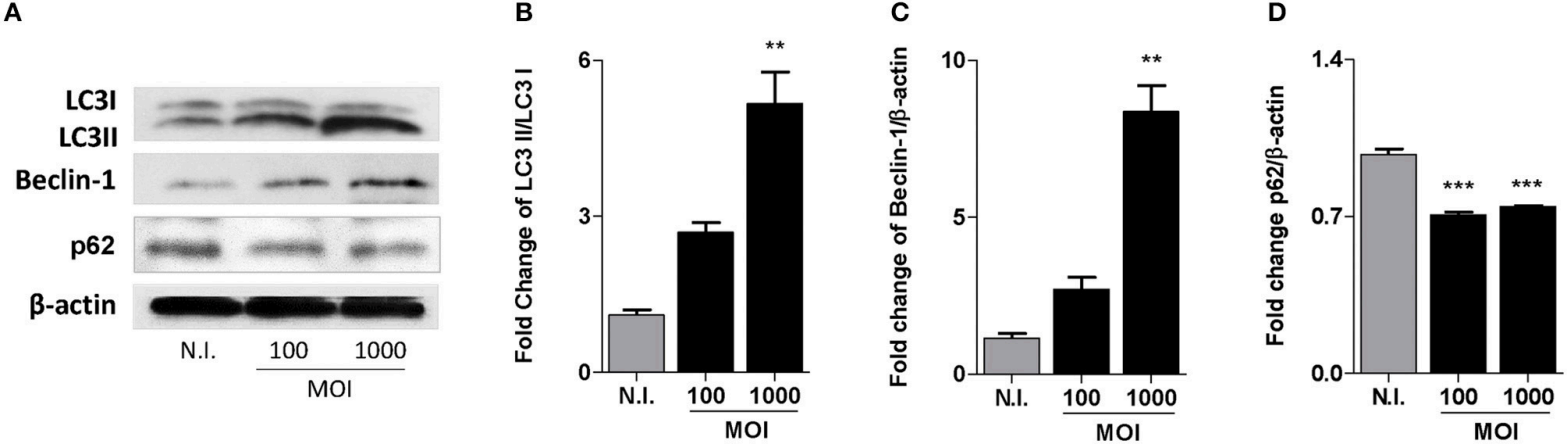
*B. abortus* infection induces autophagy in MC3T3-E1 osteoblasts. **(A)** MC3T3-E1 osteoblasts were infected with *B. abortus* at a MOI of 100 and 1,000, cell lysates obtained at 24 h post-infection were used to determine LC3I and II, Beclin-1, and p62 production by western blotting. **(B–D)** Densitometric analysis of results from three independent experiments performed as described for **(A)** LC3II/LC3I ratio **(B)** and Beclin-1 **(C)** and p62 **(D)** production. Data are given as the means ± SD from at least three individual experiments. ^**^P < 0.01; ^***^P < 0.001 vs. non-infected cells (N.I.).

Bone remodeling is a strongly restricted mechanism in which osteoblasts are the cells involved in bone formation. Autophagy modulate osteoblast differentiation and mineralization (Liu et al., 2013; Nollet et al., 2014). To determine if *B. abortus* infection induces autophagy in osteoblast cells we evaluated the expression of LC3II, the lipidated form of LC3I and the only known protein that specifically associate with autophagosomes (Shintani and Klionsky, 2004); the autophagy regulator Beclin-1 (He and Levine, 2010); and p62, that participates in autophagic clearance of ubiquitylated proteins (Lippai and Low, 2014). MC3T3-E1 cells were infected with *B. abortus* for 2 h and then washed to remove uninternalized bacteria; after 24 h, cells were lysed to evaluate the expression of LC3, Beclin-1 and p62 by western blotting. *B. abortus* infection induced an increase in the expression of LC3II and Beclin-1; and the inhibition of p62 expression in MC3T3-E1 cells (Figure 1). These results indicate that autophagy was activated in *B. abortus* infected MC3T3-E1 cells.

### Inhibition of Deposition of Bone Matrix Induced by *B. abortus* Infection in Osteoblast Is Dependent on Autophagosome-Lysosome Fusion, PI3-Kinase, and Lysosomal Proteases

Osteoblasts are the cells responsible for the deposition of bone matrix and are thought to assist the calcification and mineralization of the bone matrix. Increasing evidence support that autophagy is concerned in osteoblast differentiation (Liu et al., 2013; Ozeki et al., 2016; Weng et al., 2018).

**Figure 2:**
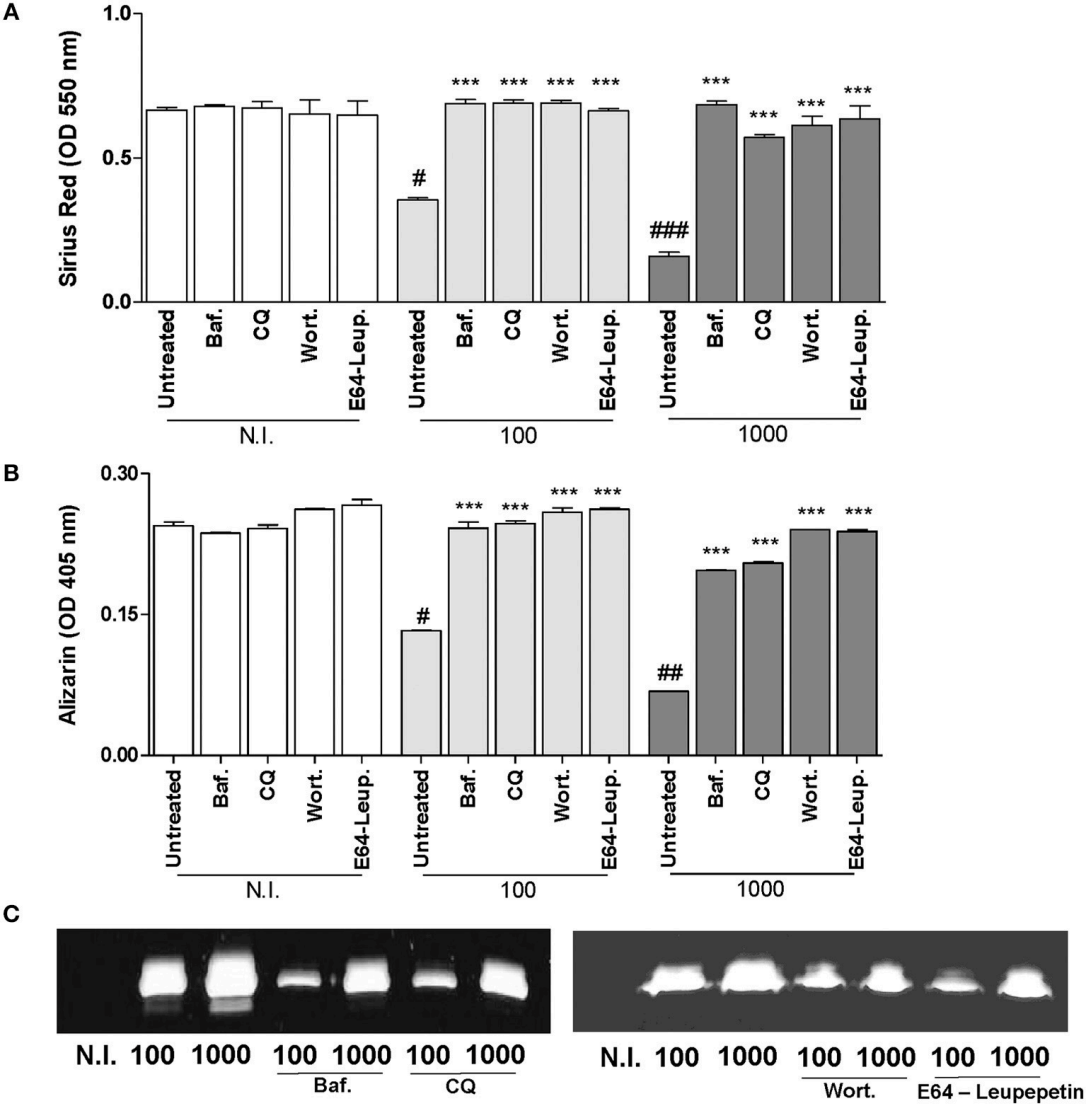
*B. abortus* inhibition of bone matrix deposition and induction of MMP-2 secretion is dependent on autophagosome-lysosome fusion, PI3-kinase and lysosomal proteases. **(A)** Effect of bafilomycin (Baf.), chloroquine (CQ), wortmannin (Wort.), and E64 plus leupeptin (E64+leup.) on the inhibition of collagen deposition during *B. abortus* infection, as determined by quantification of Sirius red staining 10 days after infection. Baf., CQ, Wort., E64+leup. Effect on calcium deposition was revealed by quantification of alizarin red S staining at 10 days post-infection **(B)**. Baf., CQ, Wort., E64+leup. Effect on MMP-2 secretion determined by zymography at 24 h after infection **(C)**. Data are given as the means ± SD from at least three individual experiments. ^***^P < 0.001 vs. infected and untreated cells. #*P* < 0.05; ##*P* < 0.01; ###*P* < 0.001 vs. non-infected (N.I.) and untreated cells.

We have demonstrated that *B. abortus* infection inhibits organic and mineral matrix deposition by osteoblast (Scian et al., 2012). Thus, experiments were conducted to determine whether inhibition of organic and mineral matrix deposition by *B. abortus* infection is dependent on the induction of the autophagy pathway. To this end, we examined markers of collagen (Sirius red staining) and mineral matrix deposition (Alizarin red S staining) during *B. abortus* infection in the presence of bafilomycin A1 or chloroquine, two inhibitors of autophagosome-lysosome fusion, wortmannin (a PI3-kinase inhibitor), and leupeptin plus E64 (inhibitors of lysosomal proteases). All inhibitors used were able to reverse *B. abortus*-induced collagen and mineral matrix deposition down-modulation (Figures 2A,**B**). *Brucella abortus* infection not only inhibited deposition of bone matrix by osteoblast, but also contributes to bone loss by the induction of MMP-2 secretion by infected osteoblast. MMP-2 secretion was also dependent on the autophagy pathway activation, since when infection experiments were performed in the presence of bafilomycin A1 or chloroquine, MMP-2 secretion was partially inhibited (Figure 2).

Taken together, these results indicate that *B. abortus* infection modulates osteoblast function through a mechanism that is dependent on autophagosome-lysosome fusion, PI3-kinase activation and lysosomal proteases.

### *B. abortus* Intracellular Replication Is Not Necessary to Inhibit Osteoblast Function

**Figure 3:**
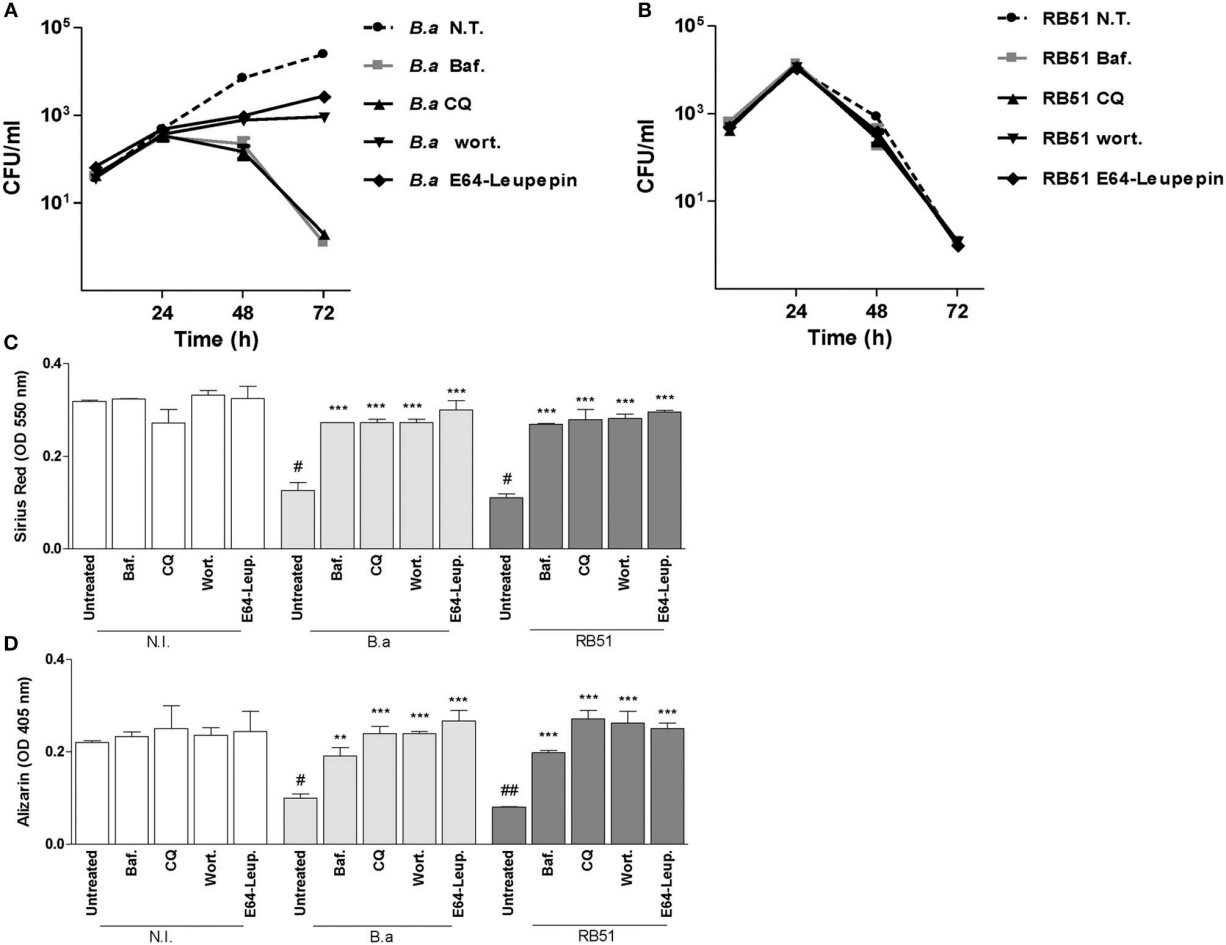
*B. abortus* intracellular replication is not necessary to inhibit osteoblast function. MC3T3-E1 osteoblasts were infected with *B. abortus* (*B. a*), *B. abortus* RB51 (RB51) at MOI 1,000, and CFU levels were determined at 4, 24, 48, and 72 h post-infection **(A,B)**. Effect of bafilomycin (Baf.), chloroquine (CQ), wortmannin (Wort.), and E64 plus leupeptin (E64+leup.) on the inhibition of collagen deposition assessed by quantification of Sirius red **(C)** and calcium deposition revealed by quantification of alizarin red S staining **(D)** at 10 days during *B. a* and RB51 infection. N.T.: non-treated. Data shown are from a representative experiment of three performed. ^**^P < 0.01; ^***^P < 0.001 vs. infected and untreated cells. #*P* < 0.05; ##*P* < 0.01; vs. non-infected (N.I.) and untreated cells.

Autophagy-associated proteins are required for *B. abortus* replicative vacuole biogenesis in macrophages, the main replication niche for *Brucella* (Starr et al., 2012; Celli, 2015). Thus, we decided to determine the role of autophagy in *B. abortus* replication in osteoblast. To this end, infection experiments were conducted in the presence of bafilomycin A1, chloroquine, wortmannin, and leupeptin plus E64. Our results indicated that bafilomycin A1, chloroquine, wortmannin, and leupeptin plus E64 inhibit *B. abortus* intracellular replication in osteoblasts (Figure 3). This could indicate that the reversion of the effect of *B. abortus* on osteoblast function could be dependent on bacteria replication.

To determine whether intracellular replication is critical for inhibition of matrix deposition and for induction of MMP-2 secretion, experiments were performed using the RB51 rough mutant. As occurs in other cell types (Rittig et al., 2003) *B. abortus* RB51 was unable to replicate in osteoblast cells. However, *B. abortus* RB51 infection inhibits collagen deposition and mineral matrix deposition, and the phenomena was also dependent on autophagy pathway, since the effect was reversed when infection experiments were performed in the presence of bafilomicyin A1, chloroquine, wortmannin, and leupeptin plus E64 (Figure 3). These results indicated that inhibition of matrix deposition was not dependent on intracellular replication, but is dependent on autophagosome-lysosome fusion, PI3-kinase, and lysosomal proteases.

### Inhibition of Autophagy at 5 Days Post-infection Does Not Reverse the Inhibitory Effect on Osteoblast Function Induced by *B. abortus* Infection

**Figure 4:**
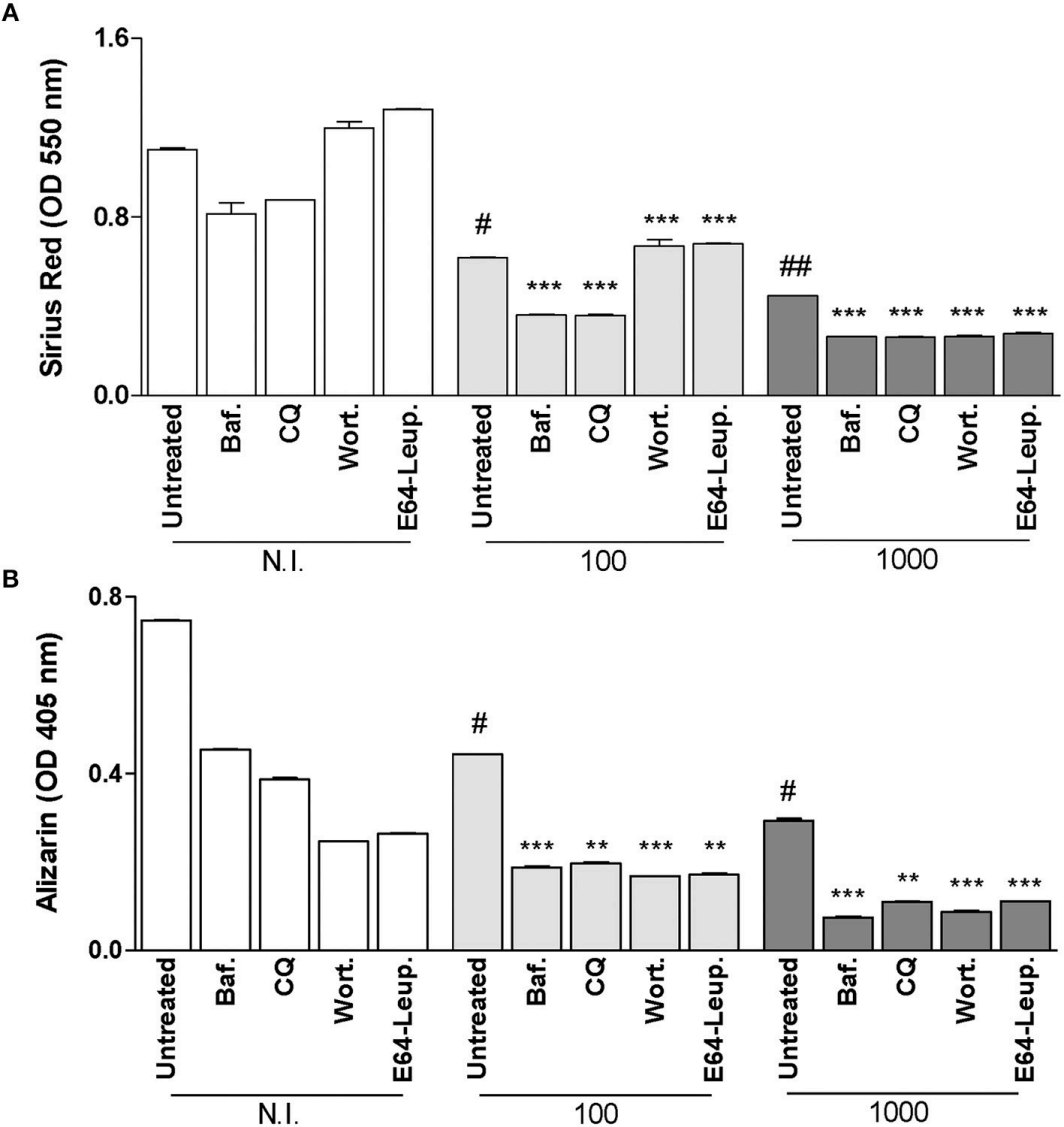
Inhibition of autophagy at 5 days post-infection does not reverse the inhibitory effect on osteoblast function induced by *B. abortus* infection. **(A)** Effect of bafilomycin (Baf.), chloroquine (CQ), wortmannin (Wort.), and E64 plus leupeptin (E64+leup.) on the inhibition of collagen deposition during *B. abortus* infection, as determined by quantification of Sirius red staining 10 days after infection. Baf., CQ, Wort., E64+leup. Effect on calcium deposition was revealed by quantification of alizarin red S staining at 10 days post-infection **(B)**. Data are given as the means ± SD from at least three individual experiments. ^**^P < 0.01; ^***^P < 0.001 vs. infected and untreated cells. #*P* < 0.05; ##*P* < 0.01; vs. non-infected (N.I.) and untreated cells.

It has been demonstrated that autophagic process is required during later stages of osteoblast differentiation and mineralization process (Nollet et al., 2014). Then experiments were conducted to determine the role of autophagy during *B. abortus* infection by adding bafilomycin A1, chloroquine, wortmannin, and leupeptin plus E64 between day 5 and 10 of osteoblast differentiation. Inhibition of autophagosome-lysosome fusion by the inhibitors bafilomycin A1 and chloroquine, the PI3-kinase inhibitor wortmannin and inhibitors of lysosomal proteases leupeptin plus E64 at 5 days post-infection could not reverse the inhibitory effect on osteoblast function induced by *B. abortus* infection (Figures 4A,**B**). In addition, inhibitors added at 5 days post the initiation of differentiation process, inhibited mineral matrix deposition also in uninfected cells (Figure 4B). This could indicate that autophagy is necessary in later stages of differentiation, and the inhibition of autophagosome-lysosome fusion, PI3-kinase, and lysosomal proteases inhibits organic and mineral matrix deposition during *B. abortus* infection.

### *B. abortus* Increases RANKL Secretion Independently of the Autophagy Pathway

**Figure 5:**
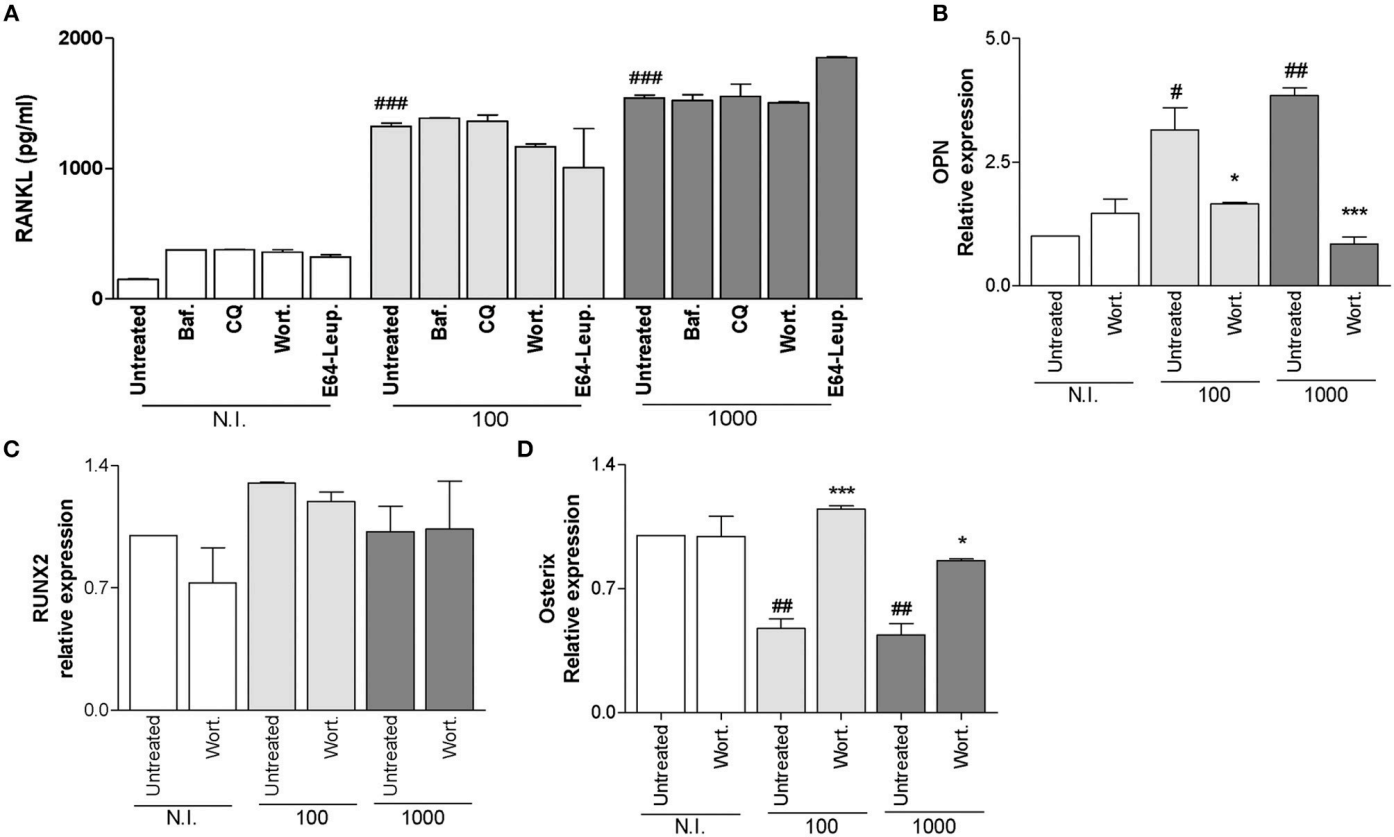
Modulation of receptor activator of nuclear factor-κB ligand (RANKL) secretion, osteopontin (OPN) and the transcription factors Runt-related transcription factor 2 (Runx2) and osterix (Osx) during *B. abortus* infection. **(A)** Effect of bafilomycin (Baf.), chloroquine (CQ), wortmannin (Wort.), and E64 plus leupeptin (E64+leup.) in the secretion of RANKL induced by *B. abortus* infection by ELISA at 48h post-infection. Effect of Wort. during *B. abortus* infection on OPN **(B)**, RUNX2 **(C)**, and Osx **(D)** by RT-qPCR at 24 h post-infection. Data are given as the means ± SD from at least three individual experiments. ^*^P < 0.05; ^***^P < 0.001 vs. infected and untreated cells. #*P* < 0.05; ##*P* < 0.01; ###*P* < 0.001 vs. non-infected (N.I.) and untreated cells.

RANKL is a key molecule implicated in bone remodeling under physiological conditions (Suda et al., 2001). *B. abortus* infection induces the increase in RANKL expression (Scian et al., 2012). When osteoblasts were infected with *B. abortus* in the presence of bafilomycin A1, chloroquine, wortmannin, and leupeptin plus E64, these cells expressed similar levels of RANKL respect to those expressed by infected and untreated cells (Figure 5A). These results indicate that inhibition of autophagosome-lysosome fusion, PI3-kinase, and lysosomal proteases has no effect on the expression of RANKL induced by *B. abortus* infection in osteoblasts.

### *B. abortus* Infection Increase Osteopontin (OPN) Expression Depending on the Authophagy Pathway

OPN is one of the abundant non-collagenous proteins in bone matrix. Several studies reported that OPN expression is increased in bone tissues of chronic bone inflammatory diseases such as rheumatoid arthritis, periodontitis, and metastatic cancer in bone (Kido et al., 2001; Ohshima et al., 2002; Carlinfante et al., 2003; Kusuyama et al., 2017).

Since autophagy pathway could be suppressed at any stage of autophagic flux and taking into account all of inhibitors used until now had similar effect; we decided to simplify the experiment by the use of wortmannin for autophagy pathway inhibition.

Then experiments were conducted to determine if *B. abortus* infection could modulate OPN expression in a way that is dependent on autophagy pathway.

Our results indicated that *B. abortus* infection induces OPN expression in a mechanism that requires PI3-kinase, since up-regulation was inhibited by wortmannin treatment (Figure 5B).

### Modulation of Transcription Factors Runx2 and OSX During *B. abortus* Infection

Runx2 and Osx are the master transcription factors in bone formation (Ducy et al., 1997; Komori et al., 1997; Otto et al., 1997; Nakashima et al., 2002). Then experiments were conducted to determine if *B. abortus* infection could modulate Runx2 expression and if this modulation is dependent on autophagy. Our results indicated that *B. abortus* infection was not able to modulate Runx2 expression. Additionally, the treatment with wortmannin had no effect on Runx2 expression (Figure 5C). Although it has been established that Runx2 is upstream of Osx and could regulate its expression, other studies indicate that Osx expression is also mediated through alternate pathways independently of Runx2 during osteoblast formation (Lee et al., 2003). Then experiments were conducted to evaluate if *B. abortus* infection could modulate Osx expression. *B. abortus* inhibits Osx expression, and this inhibitory effect was dependent on autophagy pathway, since when infection experiments were performed in the presence of wortmannin the inhibitory effect induced by *B. abortus* infection was completely reversed (Figure 5D).

### Bone Morphogenetic Protein (BMP)-2 Treatment Reverses the Inhibitory Effect of *B. abortus* Infection on Matrix Deposition

**Figure 6:**
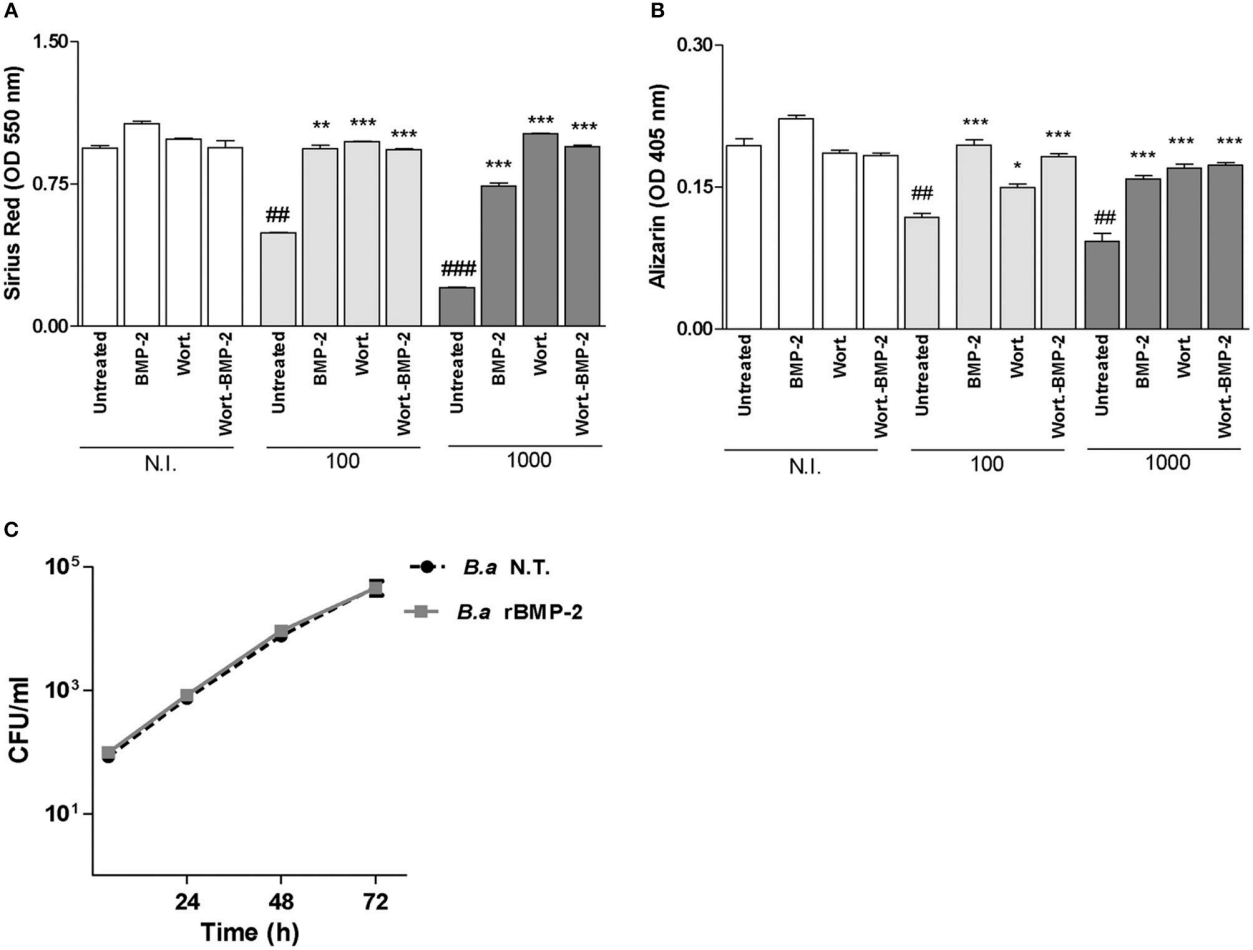
Bone morphogenic protein (BMP)-2 treatment reverses the inhibitory effect of *B. abortus* infection on matrix deposition. Effect of BMP-2 (10 ng/ml) on inhibition of collagen deposition during *B. abortus* infection, as determined by quantification of Sirius red staining at 10 days after infection **(A)**; and on calcium deposition revealed by quantification of alizarin red S staining at 10 days post-infection **(B)**. MC3T3-E1 osteoblasts were infected with *B. abortus* (*B. a*) in the presence or not of recombinant bone morphogenetic protein-2 (rBMP-2) at MOI 1,000, and CFU levels were determined at 4, 24, 48, and 72 h post-infection **(C)**. N.T.: non-treated. Data are given as the means ± SD from at least three individual experiments. ^*^P < 0.05; ^**^P < 0.01; ^***^P < 0.001 vs. infected and untreated cells. ##*P* < 0.01; ###*P* < 0.001 vs. non-infected (N.I.) and untreated cells.

Although the ability of autophagy inhibitors to reverse the inhibitory effects of mineral and organic matrix deposition during *B. abortus* infection, the use of autophagy components as molecular target for treatment during *B. abortus* infection could be controversial due to the differential effect depending on the time of infection and the time of osteoblast differentiation. BMP-2 has been used as treatment for bone pathology and approved by FDA in 2002 (Walker et al., 2014), however this protein has not been studied in the context of bacterial bone damage. BMP-2 induces Osx in osteoblast independently of the levels of Runx2 activity, with the induction of bone matrix deposition (Celil et al., 2005). Then experiments were conducted to determine if BMP-2 treatment could reverse the inhibitory effect of *B. abortus* on matrix deposition. To this end, we examined markers of osteoblast differentiation during *B. abortus* infection in the presence of rBMP-2. Our results indicated that rBMP-2 was able to reverse *B. abortus*-induced collagen and mineral matrix deposition down-modulation (Figures 6A,**B**). In addition rBMP-2 had no effect on *B. abortus* intracellular replication (Figure 6C). Taken together these results indicate that the inhibition of mineral and organic matrix deposition during *B. abortus* infection could also be reversed by the treatment with rBMP-2.

## Discusion

Human brucellosis is characterized by non-specific complaints, such as backache and joint pains that can resemble acute rheumatic fever. *Brucella* localizes in different tissues of the body, and osteoarticular brucellosis is the most common form of active disease (Young, 1989; Madkour, 2001).

Bone homeostasis is dependent on the coordinated action of osteoblast, osteocytes and osteoclast. This process involved, in part, autophagy protein degradation, indicating that this cellular pathway is crucial for development, differentiation, survival of bone cells, and bone homeostasis (Shapiro, 1999; Hocking et al., 2012; Nollet et al., 2014). Its implication in human diseases has been highlighted in recent years (Jiang and Mizushima, 2014). Studies in bone suggest that dysregulation of autophagy may also be associated with the process of bone loss and subsequent osteoporosis (Pierrefite-Carle et al., 2015). In osteoblast, several data show that autophagy is involved in major aspects of differentiation and function as cells involved in mineral and organic matrix deposition (Nollet et al., 2014).

Our results indicated that *B. abortus* infection induces an increase in the levels of beclin-I and LC3-II protein, but inhibits the course of matrix deposition in osteoblast. Inhibition of autophagy pathway with the inhibitors of autophagosome-lysosome fusion (bafilomycin A1 and chloroquine), the PI3-kinase inhibitor (wortmannin), and the inhibitors of lysosomal proteases (leupeptin plus E64), at the early time of differentiation reverses the inhibitory effects on matrix deposition induced by *B. abortus* infection. This result appear to be contradictory, since it was demonstrated that autophagy increases during osteoblast differentiation and mineralization (Nollet et al., 2014). However, it has been demonstrated that autophagy was required for osteoblast terminal differentiation (Liu et al., 2013). In this context, infection experiments were performed by added autophagy inhibitors at day 5 post-infection, and in this case our results indicated that the inhibitors, not only fails to reverse the inhibitory effect of *B. abortus* infection on organic and mineral matrix deposition, but also uninfected cells treated with autophagy inhibitors deposited less matrix than their untreated controls. This indicates that *Brucella* infection uses the autophagic pathway to inhibit matrix deposition early during infection, while at later times the process of differentiation of osteoblasts takes control of the pathway.

In addition to its role as a cell involved in depositing bone matrix, osteoblast also expresses RANKL. It is a key molecule implicated in bone remodeling. The primary role of RANKL is the induction of osteoclastogenesis, once expressed on osteoblasts, RANKL initiates bone resorption (Takayanagi, 2010). Moreover, RANKL is upregulated in bacterial osteomyelitis contributing to bone damage (Claro et al., 2011).

Previous studies performed in calvaria from *Atg5* deficient mice associated autophagy deficiency with an increase of RANKL secretion (Nollet et al., 2014). Our results indicated that inhibition of autophagy with pharmacological inhibitors induce an increase in the secretion of RANKL that was not statistically significant. Additionally, inhibition of autophagy had no effect on the secretion of RANKL in infected cells respect to infected and untreated controls. This indicates that the autophagic pathway is not a good target to modulate the expression of RANKL during the pathological increase induced by *Brucella* infection.

OPN is one of the non-collagenous proteins present in bone matrix. The precise roles of OPN in osteoblastic functions in non-pathological conditions remain ambiguous (Kusuyama et al., 2017). However, numerous studies reported that OPN expression is increased in the bone tissues of patients with chronic bone inflammatory diseases such as rheumatoid arthritis, periodontitis, and metastatic cancer in bone (Kido et al., 2001; Ohshima et al., 2002; Carlinfante et al., 2003). But, their role in infectious bone pathology has not been described yet. *B. abortus* infection induces OPN expression, suggesting that OPN might exert negative effects on osteoblastic function not only in sterile inflamed bone tissue but also in bacterial infected tissue. The induction of OPN by *B. abortus* was dependent on autophagy as was revealed by the use of wortmannin. The interaction between OPN expression and autophagy was previously associated with osteoblast differentiation, when it was demonstrated that inhibition of autophagy with chloroquine, inhibited OPN expression, that was increased when cells were treated with the autophagy inductor rapamycin (Ozeki et al., 2016).

Runx2 and Osx are the master transcription factors in bone formation (Ducy et al., 1997; Komori et al., 1997; Otto et al., 1997; Nakashima et al., 2002).

*Brucella abortus* infection was not able to modulate Runx2 expression. Autophagy inhibition by using wortmannin was unable to modulate Runx2 expression in infected and uninfected cells. The role of autophagy in Runx2 expression was previously study by others and although the results are controversial, some author reported that osteoblast from calvaria from Atg5 deficient mice express higher levels of Runx2 that control osteoblast genes involved in mineralization of bone matrix deposition (Nollet et al., 2014). Atg5 deficient MC3T3-E1 cells express lower levels of Runx2 and the over expression of Atg5 did not modulate the levels of Runx2 expression respect the control cells (Weng et al., 2018). May be these discrepancies could be attributed to the fact that the changes induced in both increase or reduction of the expression are < 1-fold of increase which introduces a lot of error due to small experimental changes.

As it was mentioned Osx is also involved in regulation of osteoblast differentiation, and our results indicated that *B. abortus* infection inhibits Osx expression and this inhibition was reversed when infection experiments were performed in the presence of wortmannin. It has been determined that Osx is downstream of Runx2 gene (Nakashima et al., 2002). But, in concordance with our results, other studies indicate that Osx expression is also mediated through alternate pathways independently of Runx2 during osteoblast formation (Lee et al., 2003).

Our results indicate that the autophagic pathway might be a target for the treatment of infectious osteomyelitis. However, the reversal induced by BMP-2 of the inhibition of bone matrix deposition induced by *Brucella* infection, would indicate that it could also be considered as co-treatment along with antibiotic therapy.

## Author Contributions

AP and MD conceived, designed the experiments and interpreted the data. AP performed the experiments and analyzed the data. MG assisted in cell culture. PA assisted technically in the realization of western blotting. GG supported the work with key suggestions. MD supervised experiments and wrote the manuscript. All authors reviewed the manuscript.

### Conflict of Interest Statement

The authors declare that the research was conducted in the absence of any commercial or financial relationships that could be construed as a potential conflict of interest.
